# Innovative Therapies Targeting Drug-Resistant Biomarkers in Metastatic Clear Cell Renal Cell Carcinoma (ccRCC)

**DOI:** 10.3390/ijms26010265

**Published:** 2024-12-31

**Authors:** Moses Owoicho Abah, Deborah Oganya Ogenyi, Angelina V. Zhilenkova, Freddy Elad Essogmo, Yvan Sinclair Ngaha Tchawe, Ikenna Kingsley Uchendu, Akaye Madu Pascal, Natalia M. Nikitina, Alexander S. Rusanov, Varvara D. Sanikovich, Yuliya N. Pirogova, Alexander Boroda, Aleksandra V. Moiseeva, Marina I. Sekacheva

**Affiliations:** 1 World-Class Research Center “Digital Biodesign and Personalized Healthcare”, Sechenov First Moscow State Medical University, Moscow 119991, Russia; ogenyideborah@gmail.com (D.O.O.); av.zhilenkova@gmail.com (A.V.Z.); essogmo_f@student.sechenov.ru (F.E.E.); ngakhatchave_i@student.sechenov.ru (Y.S.N.T.); akayepaschal48@gmail.com (A.M.P.); nikitinanm@gmail.com (N.M.N.); alexrus146@yandex.ru (A.S.R.); v8varvara@gmail.com (V.D.S.); pirogova_yuliya96@mail.ru (Y.N.P.); alexcom90@yandex.ru (A.B.); moiseeva_a_v@student.sechenov.ru (A.V.M.); sekach_rab@mail.ru (M.I.S.); 2 Department of Cancer Bioinformatics and Molecular Biology, Royal Society of Clinical and Academic Researchers (ROSCAR) International, Abuja 900104, Nigeria; 3 Medical Laboratory Science Department, Faculty of Health Science and Technology, College of Medicine, University of Nigeria, Enugu Campus, Enugu 410001, Nigeria

**Keywords:** biomarkers, drug resistance, immunotherapies, targeted therapies, ccRCC, metastasis

## Abstract

A thorough study of Clear Cell Renal Cell Carcinoma (ccRCC) shows that combining tyrosine kinase inhibitors (TKI) with immune checkpoint inhibitors (ICI) shows promising results in addressing the tumor-promoting influences of abnormal immunological and molecular biomarkers in metastatic Clear Cell Renal Cell Carcinoma (ccRCC). These abnormal biomarkers enhance drug resistance, support tumor growth, and trigger cancer-related genes. Ongoing clinical trials are testing new treatment options that appear more effective than earlier ones. However, more research is needed to confirm their long-term safety use and potential side effects. This study highlights vital molecular and immunological biomarkers associated with drug resistance in Clear Cell Renal Cell Carcinoma (ccRCC). Furthermore, this study identifies a number of promising drug candidates and biomarkers that serve as significant contributors to the enhancement of the overall survival of ccRCC patients. Consequently, this article offers pertinent insights on both recently completed and ongoing clinical trials, recommending further toxicity study for the prolonged use of this treatment strategy for patients with metastatic ccRCC, while equipping researchers with invaluable information for the progression of current treatment strategies.

## 1. Introduction

Renal cell carcinoma (RCC) is the most prevalent form of adult kidney cancer. RCC symptoms may include fever, weight loss, hypertension, and hematuria [1,2,3,4,5,6]. Progressive modalities like immunotherapy present promise for prolonged survival. RCC are 96% sporadic and 4% familial. Its common risk factors include smoking, excessive alcohol consumption, overweight, high blood pressure, exposure to reno-toxic chemicals like trichloroethylene, and chronic kidney failure. Studies show that low alcohol consumption and an omega 3-rich fatty fish diet and diet rich in fruit/vegetables prevent RCC [7,8,9,10,11,12]. The management of RCC depends significantly on the tumor stage. Treatment modalities vary from surgical procedures, such as nephrectomy or partial nephrectomy, to non-surgical approaches like radiation therapy, cryotherapy, immunotherapy, localized radiation therapy, or targeted therapy for metastasis. In cases where RCC induces varicocele by impeding the blood flow through the renal vein, the sole remedy is the surgical excision of the RCC.

Clear Cell Renal Cell Carcinoma (ccRCC) is a prevalent urological subtype of renal cell carcinoma (RCC), posing a significant challenge in the realm of oncology. The World Health Organization (WHO) classifies RCC into clear cell RCC, papillary RCC, chromophobe RCC, hereditary leiomyomatosis RCC, the multilocular cystic renal neoplasm of low malignant potential, acquired cystic disease-associated RCC, tubulocystic RCC, clear cell papillary RCC, mucinous tubular and spindle cell carcinoma, renal medullary carcinoma, collecting duct carcinoma, succinate dehydrogenase (SDH)-deficient RCC, and MiT Family translocation carcinoma, alongside other unclassified types of RCC [13]. ccRCC makes up the majority of cancer-related deaths and differs from other RCC types like papillary and chromophobe RCC [13]. It also represents the largest share of RCC cases, while less common types include renal medullary carcinoma, renal sarcomas, collecting duct tumors, Wilms’ tumors, and nephroblastoma [14]. ccRCC often remains clinically occult, lacking severe symptoms, the median age at diagnosis is around 60 years old, and it is more prevalent in men than women and also more common in certain racial groups other than the Asian population [15,16].

Clear Cell Renal Cell Carcinoma (ccRCC) is predominantly caused by a mutation in tumor suppressor–molecular biomarkers. For example, the loss of the 3p chromosome arm disrupts critical genes like VHL, PBRM-1, BRCA1, and MTOR that act as tumor suppressors. A pathogenic variant of these genes leads to a loss of function, favoring cell proliferation and tumorigenesis [17,18,19,20,21,22]. The research underscores miR-20b-5p as a promising target, while immunological markers such as CTLA4, PD-1, and PD-L1 are linked to poorer prognosis. Despite uncertainty surrounding BTLA’s impact, there is optimism regarding CD40, GITR, and OX40 for improved outcomes [23,24,25,26,27].

The ccRCC treatment landscape entails a fusion of targeted therapies and immunotherapies to combat drug resistance. A thorough comprehension of these markers and checkpoint proteins is imperative for personalized treatments and renewed optimism in tackling ccRCC [28,29,30,31,32,33,34,35,36]. Targeting these growth factors and pathways holds promise for more effective therapies [37,38,39,40]. A comprehensive understanding of the mechanism of action of these immune checkpoint proteins and molecular markers associated with ccRCC will greatly contribute to the development of novel targeted therapies and immunotherapies [41,42,43]. These therapies may function by reactivating the immune system, inhibiting or activating these tumor-promoting or -suppressing growth factors as well as their receptors and tumor-promoting pathways, alongside the downregulation of the various oncogenes activated in their signal transduction axes [44,45,46,47].

Clear cell renal cell carcinoma (ccRCC) is a type of kidney cancer that has been extensively studied, and significant advancements have been made in understanding how it develops and how to treat it. However, a major problem that remains is the ability of ccRCC to become resistant to treatments over time. This resistance is a serious issue because it limits the effectiveness of current therapies and makes it harder to control the cancer.

One of the main challenges is that ccRCC can resist treatment in two ways: intrinsic resistance, which means the cancer does not respond to treatment from the start, and acquired resistance, which develops after an initial period when the treatment seemed to work. Both types of resistance often lead to poor outcomes for patients, especially when treated with tyrosine kinase inhibitors (TKIs), a common type of cancer therapy.

The mechanisms behind this resistance are complex, but generally involve changes in the tumor’s environment. For example, the tumor microenvironment—the area surrounding the tumor—can adapt to make the cancer harder to treat. Additionally, the cancer can activate hypoxia-induced factors, which are proteins that help it survive in low-oxygen conditions. It can also upregulate, or increase, alternative pathways that promote the growth of new blood vessels (angiogenesis), ensuring the tumor continues to receive nutrients and oxygen despite treatment.

These adaptations make ccRCC a challenging cancer to treat and highlight the need for continued research to develop more effective therapies that can overcome or prevent resistance.

## 2. Insight on Clear Cell Renal Cell Carcinoma (ccRCC)

### 2.1. Metastatic ccRCC: Pathological Characteristics and Recent Treatment Advances

Metastatic clear cell renal cell carcinoma (ccRCC) is a highly aggressive form of ccRCC characterized by significant heterogeneity and a complex tumor microenvironment, which present substantial obstacles to its effective treatment and management. Despite the development of various therapeutic strategies, diagnosing and treating metastatic ccRCC remains a major challenge. However, recent advancements in combining targeted therapies with immunotherapy have significantly improved its management and treatment outcomes.

#### 2.1.1. Immunotherapy and Combination Therapy

The use of immune checkpoint inhibitors has played a pivotal role in the safe and effective management of metastatic ccRCC, offering new hope for patients [48]. Combining immune checkpoint inhibitors with other therapeutic approaches via combination therapy has significantly enhanced the overall survival rates in patients with metastatic ccRCC, marking a critical advancement in treatment strategies [48].

#### 2.1.2. Prognostic Biomarkers

Recent studies have highlighted elevated circulating tumor cell levels as an indicator of poor survival outcomes in patients. This emerging prognostic biomarker marks a significant advancement in accurately predicting the ccRCC response to therapy [49]. Additionally, a resting mast panel was recently developed to enhance patient prognosis by aiding in the prediction of distant metastasis and assessing responses to treatment strategies [50].

#### 2.1.3. Tumor Microenvironment and Molecular Insights

Single-cell RNA sequencing studies have revealed links between tumor states associated with metastasis and specific immune cell compositions. These findings pave the way for developing personalized therapeutic strategies for metastatic ccRCC [51,52].

### 2.2. Genetic Mutations and Molecular Markers in Clear Cell Renal Cell Carcinoma (ccRCC)

#### 2.2.1. ccRCC Genetic Mutations

It is noteworthy that ccRCC biomarkers, which typically function as tumor suppressors under normal conditions, can take on a different, more nefarious role when they become pathological. The genesis of these defects often stems from specific genetic alterations [53]. Notably, these alterations include a gain of chromosome 5q, accounting for a substantial 69% of ccRCC cases, a gain of chromosome 7q in over 20% of cases, a partial loss of chromosome 14q in more than 42% of cases, a deletion of chromosome 8p found in 32% of cases, a loss of the 9p chromosome in 29% of cases, and a complete loss of the 3p chromosome observed in a striking 95% of cases [54].

The complex interplay of genetic mutations and their implications in ccRCC is a subject of paramount importance. While these molecular markers normally serve to keep cancer at bay, it is fascinating and somewhat alarming to see how they can become key players in promoting tumorigenesis when they undergo genetic alterations. This shift in their function is often the result of specific chromosomal aberrations, which are highly prevalent in ccRCC cases [51,52,53,54].

#### 2.2.2. Exploring the Significanceof VHL Molecular Marker in ccRCC

In ccRCC, the von Hippel-Lindau (VHL) molecular marker takes center stage, playing a pivotal role that shapes our understanding of this complex cancer. VHL operates as a tumor suppressor, diligently guarding against tumorigenesis in ccRCC. When functioning normally, it orchestrates a remarkable downregulation of proliferation activities, acting as a potent inhibitor of tumor progression. However, genetic alterations in VHL, often linked to the loss of chromosome 3p, compromise its tumor-inhibitory role [55]. These genetic alterations manifest in two distinct forms: inherited, as an autosomal dominant trait marked by the deletion or inactivation of one of the two gene copies, and sporadic, involving the somatic inactivation or deletion of both gene copies, known as alleles [55,56].

Interestingly, inherited pathogenic variants of the VHL gene have a high penetrance, with 90% of the patients presenting a VHL manifestation by the age of 65. VHL disease is characterized by the presence of benign and malignant tumor formation [56,57,58,59]. At the heart of VHL’s function lies the von Hippel-Lindau protein (pVHL), a regulator of KIRC-associated proteins. Among these proteins, Hypoxia Inducible Factor 1 Alpha (HIF1A) and Hypoxia Inducible Factor 2 Alpha (HIF2A) act as conductors in a complex molecular orchestra within the cell. pVHL steps in as the diligent conductor, preventing these conductors from accumulating excessively [59,60]. 

The HIF1A-HIF2A complex favors tumorigenesis by activating other growth factors, such as Platelet-Derived Growth Factor Beta (PDGFB), Transforming Growth Factor Alpha (TGFA), and Vascular Endothelial Growth Factor (VEGF), which drive the progression of ccRCC [60]. The absence of pVHL leads to the accumulation of the HIF1A-HIF2A complex which is further potentiated by a lack of oxygen in the tumor. pVHL’s role is like a conductor ensuring the orchestra of HIF1A and HIF2A does not go overboard, thus influencing the production of growth factors and ultimately impacting the course of ccRCC tumor progression [60]. Oxygen availability emerges as a critical factor in the tumor’s progression or inhibition. Under conditions of oxygen abundance, HIF1A and HIF2A are targeted and destroyed by pVHL. However, in an oxygen deficiency, the absence of pVHL, or in the presence of abnormal pVHL, these proteins accumulate, forming the HIFB complex [60,61].

Current therapeutic strategies for ccRCC primarily revolve around tyrosine kinase-inhibitors targeting VEGF. Promisingly, research is exploring HIF2A inhibitors, with compounds like PT2385 displaying potent potential against metastatic ccRCC. Investigations into HIF2A inhibitors PT2977 and PT 2399 have shown effectiveness, particularly against solid ccRCC tumors [62,63].

#### 2.2.3. PBRM-1 Gene

The PBRM-1 gene encodes the BAF180 protein, a component of the nucleosome/chromatin remodeling complex that controls gene expression by interacting with DNA [63]. Although BAF180’s precise role as a tumor suppressor is not fully understood, studies have revealed that the pathogenic variant in the protein polybromo 1 (PBRM-1) gene is found in nearly 30% of RCC; about 23.2% of PBRM-1 genetic alterations occur in ccRCC, while a significant portion of the 61.4% is associated with various cancers. PBRM-1 resides on chromosome 3p21, and common mutations include splice, nonsense, and frameshift mutations [64]. BAF180 appears to play a crucial role in regulating the cell cycle, acting as an inhibitor of cell cycle promoters. For instance, it was able to halt the cell cycle in a study involving BAF180-deficient cell lines, underscoring its importance. When the PBRM-1 gene is flawed, it results in an impaired BAF180 protein, leading to uncontrolled cell cycle activities and the subsequent unchecked proliferation of cancerous cells [65].

PBRM-1 also contributes to the regulation of P21, a well-known cell cycle inhibitory protein. Its primary function is to inhibit cell cycle promoters, such as Cyclin-Dependent Kinases 1 and 2, which would otherwise promote tumor progression by driving the uncontrolled proliferation of cancer cells. PBRM-1’s role here involves binding to p53, which, in turn, activates P21. This activation prevents the CDK proteins from facilitating cellular proliferation by effectively blocking their receptors, thereby hindering tumorigenesis in ccRCC [65]. PBRM-1 and its associated BAF180 protein seem to act as gatekeepers of the cell cycle, preventing uncontrolled cell division and potentially serving as a barrier against tumor development in metastatic Clear Cell Renal Cell Carcinoma (ccRCC) [66].

#### 2.2.4. BRCA 1-Associated Protein-1 (BAP-1)

BRCA 1-Associated Protein-1 (BAP-1) is a pivotal anti-tumor protein that binds to the Breast Cancer 1 (BRCA-1) gene and plays a crucial role in deubiquitinating ubiquitin [67,68]. BAP-1 is located in chromosome 3p21 and a mutation in this chromosome could lead to a pathogenic variant of the gene, with it losing function and leading to highly aggressive ccRCC. BAP-1 functions by activating HCF-1, using its N-terminal linked to the C-terminal that has ubiquitin hydrolase. This helps BAP-1 in the breaking down of ubiquitin. In doing so, BAP-1 cooperates with other factors to exert inhibitory control over tumor cell proliferation activities [68]. Furthermore, BAP-1 contributes significantly to DNA repair by mobilizing the Polycomb Deubiquitylase complex (PR-DUB) to the site of DNA damage. In cases of DNA damage induced by factors like ultraviolet (UV) rays, BAP-1’s role extends to mobilizing Poly ADP-Ribose Polymerase 1 (PARP 1) to the damage site, enhancing the repair process through its deubiquitination function [68].

Notably, a specific glutamic acid residue at position 31 in BAP-1 is highly prone to mutation in ccRCC. This mutation results in a loss of function and subsequent tumorigenesis. The research suggests that a BAP-1 mutation is significantly influenced by its interaction with genetically altered BRAF (v-raf murine sarcoma viral oncogene homolog B1), particularly when both genes are co-expressed. When BAP-1 is functioning correctly, it acts as a crucial guardian against tumor development in ccRCC by inhibiting cell proliferation and aiding in DNA repair [68,69]. However, mutations in BAP-1, often influenced by interactions with other genes like BRAF, can lead to a loss of its tumor-suppressive functions and contribute to the development of aggressive ccRCC [69].

#### 2.2.5. MTOR Molecular Function in ccRCC

mTOR helps control the cell cycle by slowing down p21 through mTORC1, causing p21 to break down. It is fascinating that when 4E-BP1 becomes phosphorylated, it stops mTOR from attaching to p21, and this, surprisingly, makes p21 more effective at halting the cell. This discovery holds promise for cancer treatment, but requires further investigation [70]. The mTOR-PI3K pathway, crucial for ccRCC-related tumor development, is triggered when growth factors attach to cell surfaces. This activation results in the creation of two mTOR complexes, mTORC1 and mTORC2. These complexes activate essential elements in protein translation, like 4E-BP1 and P70S6K, through phosphorylation [70]. Once activated, P70S6K moves into the cell nucleus, where it latches onto DNA and kickstarts the expression of specific genes, including HIF1A and HIF2A. These genes serve as transcription factors, spurring the production of growth factors that encourage tumor growth, including VEGF, TGFA, and PDGFB. This process also boosts other genes linked to the extracellular matrix and erythropoietin. This research illuminates mTOR’s role in ccRCC and its potential as a target for anti-cancer treatments. However, further exploration is necessary to fully grasp its therapeutic potential [70].

### 2.3. Expression of Immune Checkpoints in Metastatic Clear Cell Renal Cell Carcinoma (ccRCC)

#### 2.3.1. CD70-CD27 Axis in ccRCC

The Cluster of Differentiation 27 (CD27) receptor, when activated by its ligand Cluster of Differentiation 70 (CD70), forms the basis for the CD70/CD27 signal transduction axis. This axis plays a significant role in immune suppression and tumorigenesis by promoting the apoptosis of immune cells like T cells and B cells, ultimately dampening the host’s immune response [71]. A study by Linnemann and his team on the inhibition of membrane-proximal t-cell receptor suggests that in Kidney metastatic clear cell carcinoma (ccRCC), the CD70/CD27 axis primarily targets Tregs, a subgroup of T cells, leading to immune evasion through the T cell pathway [72].

Experimental validation confirmed that manipulating the CD70 levels impacted ccRCC tumor cell viability, particularly in the presence of a T cell co-culture [72]. The results imply that T cells are central to mediating the ccRCC tumor cell immune escape via the CD70-CD27 signaling axis [73].

#### 2.3.2. Role of CTLA4 Axis in ccRCC

Cytotoxic T-Lymphocyte-Associated 4 (CTLA4) is an immune suppressor highly expressed in ccRCC, contributing to poor overall survival. It induces T cell exhaustion through apoptosis, leading to the destruction of B and T lymphocytes, weakening the immune response against cancer cells and foreign agents. This article identifies a potential therapeutic target for CTLA4, miR-20b-5p, which exhibits a higher expression and better prognostic value in ccRCC compared to other miRNAs. Ongoing research is exploring a combination immunotherapy approach using both ipilimumab, a monoclonal antibody that inhibits the tumor-promoting activity of CTLA4, and PD-1/PD-L1 blockers for potential effectiveness in ccRCC immunotherapy [73].

#### 2.3.3. Examining the Significance of PD-1, PD-L1, and PD-L2 in Metastatic Clear Cell Renal Cell Carcinoma (ccRCC) Prognosis and Diagnosis

PD-1, primarily situated on the surface of the Cluster of Differentiation 8 (CD8) immune cells, plays a paradoxical role as a promoter of tumors when activated by its ligands, PD-L1 and PD-L2, predominantly found on tumor cells and Antigen-Presenting Cells (APCs) [73]. Once activated, PD-1 takes on the role of an immune system suppressor by targeting and crippling immune cells, which consequently weakens the host’s immune defenses. This immune escape hinges on the evasion of immune cytotoxic and apoptotic activities that immune cells like CD8 normally carry out against tumor cells, viruses, or bacteria [73,74,75]. The bright side of this complex interplay is the development of immunotherapies designed to counteract the immune-suppressive effect of activated PD-1.

Agents like nivolumab and pembrolizumab have emerged as promising treatments for metastatic ccRCC. These antibodies function by blocking the interaction between PD-1 and its ligands, PD-L1 or PD-L2. Similarly, avelumab and atezolizumab follow the same strategy of disrupting the interplay between PD-L1 and PD-1, although further research is still in progress to fully ascertain their effectiveness in treating ccRCC [75]. Studies have uncovered that PD-L1 is notably expressed in a higher percentage of clear cell tumors compared to renal non-clear cell tumors. In an in-depth investigation examining the prognostic significance of PD-L1 expression in renal cell carcinoma (RCC), it was revealed that higher PD-L1 expression in the general population correlated with an alarming 81% elevated risk of mortality. This risk more than doubled when focusing on cases assessed using immunohistochemistry [76]. Furthermore, in patients with clear cell histology, higher PD-L1 expression translated to a 53% increase in the risk of death. Intriguingly, even in patients with metastatic disease, the analysis of PD-L1 expression on primary tumors remained a valuable prognostic indicator [76].

#### 2.3.4. Exploring BTLA’s Role in Kidney Cancer Pathology

The B and T Lymphocyte Attenuator (BTLA) belongs to the CD28 immunoglobulin family. BTLA interacts with the Herpes Virus Entry Mediator (HVEM), acting as a receptor for the Tumor Necrosis Factor (TNF). It is an essential regulator produced by various immune cells, mainly responsible for moderating the activity of anti-cancer immune cells to curtail the proliferation of cancer cells [77,78,79].

An analysis utilizing the BTLA TIMER Database uncovered an interesting trend: in Papillary Renal Cell Carcinoma (PRCC), BTLA expression is notably higher in cancer cells than in the adjacent healthy tissues [77,78,79]. This sets PRCC apart from many other cancers, such as lung, bladder, colon, rectal, and thyroid carcinomas and adenocarcinomas, where BTLA tends to be downregulated and expressed at lower levels in cancer cells compared to their healthy counterparts. This unique expression pattern suggests that BTLA mRNA levels are significantly elevated in PRCC, positioning it as a valuable marker for diagnosing and predicting Papillary Renal Cell Carcinoma [80].

#### 2.3.5. Analyzing CD40/CD40L and OX4O/OX40L Expression in Metastatic Clear Cell Renal Cell Carcinoma (ccRCC)

The Cluster of Differentiation 40 (CD40) is a member of the Tumor Necrosis Factor (TNF) family activated by its ligand, the Cluster of Differentiation 40 Ligand (CD40L). It is an inflammatory protein expressed on tumor cells and Antigen-Presenting Cells (APCs). CD40 aids in the proliferation of innate immune cells like Natural Killer (NK) cells and adaptive immune cells, including B and T lymphocytes. While CD40 exhibits lower expression in renal cell carcinoma solid tumors compared to pancreatic cancer, non-small-cell lung cancer (NSCLC), and ovarian cancer, its specific role in ccRCC remains relatively unexplored [80,81,82].

An interesting finding links CD40 expression with several genes and factors, such as the C-X-C Motif Chemokine Ligand (CXCL10), Intracellular Adhesion Molecule (ICAM1), Transforming Growth Factor Beta 1 (TGFB1), Toll-Like Receptors 1, 2, and 5 (TLR1, TLR2, and TLR5), T cells, and monocytes. This study revealed this connection, although various patient-related factors, including age, disease onset, family cancer history, menopausal status at disease onset, adjuvant chemotherapeutic treatment, or disease-free survival, seemed unrelated. The presence of CD40 in the nucleus, while not associated with clinicopathological data, was more prevalent in cancer patients (83% vs. 30% in normal tissue) [83]. OX40, also known as the Cluster of Differentiation 134 (CD134), exerts tumor-suppressive effects through its role in activating T cells. Researchers are currently investigating the efficacy of OX40 agonist co-administration with PD-1 antagonist immunotherapy [83,84].

#### 2.3.6. Analyzing GITR Expression in Metastatic ccRCC

GITR, also known as the Glucocorticoid-induced Tumor Necrosis Factor Receptor (TNFRSF18) or Cluster of Differentiation 357 (CD357), is a pro-inflammatory protein activated primarily by its counterpart, the Glucocorticoid-Induced Tumor Necrosis Factor Receptor Ligand (GITRL) [85,86]. When GITR is activated, it steps in as a supportive element for B and T lymphocytes within the immune system. Its crucial role lies in preventing various forms of immune suppression, particularly by stopping the upregulation of regulatory T cells (Tregs). This action leads to the elimination of Tregs through apoptosis, thanks to GITR’s cytotoxic capabilities. In studies involving mice, the potency of DTA-1 as a GITR agonist has been evident in the treatment and management of esophageal cancer [87,88,89]. Research findings from Krausz [88,89] shed light on GITR’s distinct expression patterns.

GITR is significantly expressed in Tregs, while its presence is notably lower in naїve and memory T cells. The regulation of GITR expression is a cell-specific process, driven by FoxP3 and NF-B signaling, which upregulate GITR in mature Tregs and T cells, respectively [88,89]. Crucially, GITR’s impact extends to activated innate immune cells. Among these, activated natural killer cells exhibit the highest induction of GITR, and their GITR expression levels are comparable to those of activated T effector (Teff) cells. Dendritic cells (DCs) and activated macrophages show intermediate levels of GITR expression, while activated Tregs display the highest GITR expression [89,90,91].

### 2.4. Novel Advances in Metastatic ccRCC Therapy

#### 2.4.1. Latest Developments in First-Line KIRC Molecular and Immunotherapy Trials

**Figure 1:**
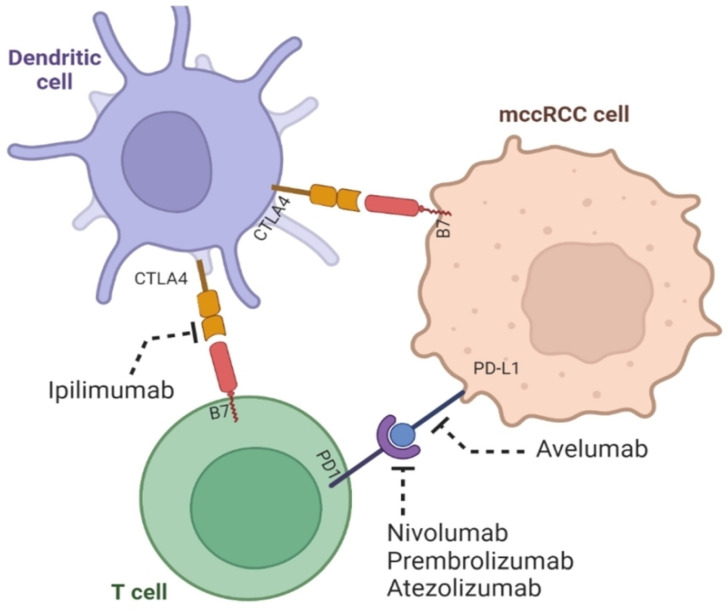
Monotherapy in tumor microenvironment of ccRCC. Checkpoint inhibitors like nivolumab, pembrolizumab, atezolizumab, and avelumab work as single treatments by blocking PD-1 and PD-L1, preventing them from suppressing the immune system and targeting T cells for destruction. On the other hand, ipilimumab blocks the cancer-promoting effects of CTLA-4. While these treatments boost the ability of T cells to fight tumors, they are less effective when used as monotherapies compared to when used as combination therapies (adapted from [33] with permission under the Creative Commons Attribution 4.0 (CC BY 4.0) license).

There have been exciting new developments in the treatment of metastatic Clear Cell Renal Cell Carcinoma (ccRCC). Recent studies have shown that using a combination of immune checkpoint inhibitors (ICI) and tyrosine kinase inhibitors (TKI) can provide better results for patients. These treatments are now being used as a first choice for many people. One important study, called Keynote 426, showed how effective this combination can be. In this study, the researchers combined two drugs, pembrolizumab and axitinib, and compared their results to a single drug, sunitinib, as shown in Figure 1 and Table 1, respectively. The results show that patients receiving the combination treatment lived longer, had better control of their cancer, and were more likely to see their tumors shrink or disappear compared to those on the single drug [92]. The study revealed a big difference between the two approaches, with the combination treatment offering much better outcomes. This is a major step forward in kidney cancer care, giving patients and doctors new options that bring hope for improved survival and quality of life.

The IMmotion 151 study showed that combining two drugs, atezolizumab and bevacizumab, works better than using just one drug, sunitinib, to treat advanced kidney cancer. Patients who received the combination therapy lived longer, had better control over the spread of their cancer, and saw greater improvements in shrinking their tumors compared to those who were treated with sunitinib alone [92].

Another important study, called CheckMate 214, focused on a different combination of drugs, nivolumab and ipilimumab [92], as shown in Table 1. This study also found that the combination was more effective than sunitinib alone [92]. Patients who received nivolumab and ipilimumab lived longer, had fewer signs of their cancer getting worse, and were more likely to have their tumors shrink or disappear completely [92].

**Figure 2:**
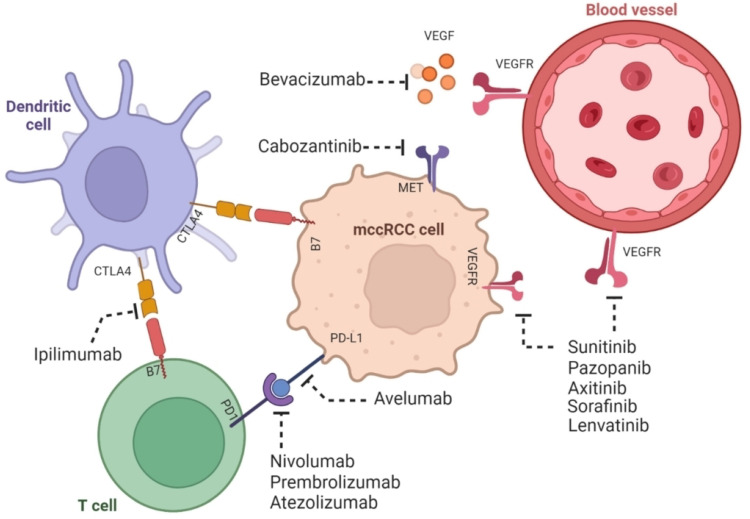
The mechanism of action of combination therapy involving tyrosine kinase inhibitors and immune checkpoint inhibitors in ccRCC is elucidated as shown. ccRCC possesses both immunogenic and angiogenic properties. Tyrosine kinase inhibitors, namely sunitinib, pazoparib, axitinib, sorafenib, lenvatinib, and cabozantinib, which fall under the category of molecular therapy, effectively impede angiogenesis (the process of new blood vessels formation). This inhibition is achieved by targeting the tumorigenic effect of growth factors such as VEGFR. Growth factors are known to contribute to the oncogenic pathway. On the other hand, the immune checkpoint inhibitors, which encompass avelumab, nivolumab, prembrolizumab, atezolizumab, ipilimumab, and bevacizumab and fall under the category of immunotherapy, exert their effectiveness by inhibiting the tumor-promoting activities of immune checkpoint proteins such as CTLA4 and PD-I. This inhibition ultimately enhances the tumor-suppressive role played by T cells and dendritic cells. (Adapted from [33] with permission under the Creative Commons Attribution 4.0 (CC BY 4.0) license.)

Patients treated with combination therapies such as pembrolizumab with axitinib, avelumab with axitinib and nivolumab with ipilimumab have shown better results and a better survival outcome compared to those who only received Sunitinib as shown in Figure 2. 

These studies are changing how doctors treat advanced kidney cancer. By combining two powerful treatments, patients are seeing much better results than with older, single-drug options. These new therapies are helping patients live longer and giving them more hope for controlling their disease. This is a big step forward in kidney cancer treatment and is expected to improve care for many people in the future.

#### 2.4.2. Assessment of Phase 3 Clinical Trials Investigating Combination Therapies for ccRCC

Table 2 summarizes new research focused on improving treatment for Clear Cell Renal Cell Carcinoma (ccRCC), a type of kidney cancer. These studies are testing combinations of two types of therapies—tyrosine kinase inhibitors (TKIs) and immune checkpoint inhibitors (ICIs)—to see how well they work when used together as a first option for treatment. The results are being compared to older, single-drug treatments to measure improvements in survival, how long the cancer is kept under control, and how well the tumors respond to treatment [93]. These trials are important because they could lead to better treatment strategies and outcomes for patients [94].

One such study, called the COSMIC 313 trial, looked at combining three drugs: cabozantinib, ipilimumab, and nivolumab. It compared this to a control group receiving just ipilimumab and nivolumab. The results showed that the three-drug combination was better at slowing down the cancer, showing that adding cabozantinib could make a difference.

Another study, the CheckMate 9ER trial, tested cabozantinib and nivolumab together against the older drug sunitinib. Patients on the combination therapy lived longer without their cancer getting worse and reported a better quality of life compared to those on Sunitinib. The CLEAR trial explored the effects of combining pembrolizumab and lenvatinib versus sunitinib. It found that the combination therapy was far more effective at controlling the cancer and improving survival. Patients on pembrolizumab and lenvatinib lived longer and had better outcomes overall than those treated with sunitinib alone.

These studies are showing that combining treatments can provide much better results for kidney cancer patients. This new approach offers hope for longer lives, better quality of life, and more effective ways to manage the disease. It is a big step forward in cancer care and is helping to shape the future of treatment for ccRCC.

## 3. Discussion and Future Directions

In ccRCC research, the future is marked by a promising horizon of innovative strategies and evolving pathways that hold great potential for revolutionizing ccRCC therapy. Delving into the intricacies of ccRCC, it is essential to review these emerging directions and explore the theoretical possibilities they offer.

The movement of the HIFA-HIFB complex from the cytoplasm to the nucleus under conditions of an oxygen deficiency is a pivotal step that promotes tumorigenesis in ccRCC [95]. The idea of preventing this complex’s translocation to the nucleus via an antagonist that induces a conformational change holds promise in inhibiting tumor growth. This approach may signify a potential breakthrough in ccRCC therapy. Understanding the roles of PD-L1 and BTLA, both in overexpression and underexpression, is pivotal in deciphering ccRCC pathology. These markers play intricate roles in the disease, and further pre-clinical research is required to unveil their potential as therapeutic targets [95,96,97,98,99,100]. To enhance the efficacy of anti PD-1/PD-L1 therapy, the combination of molecular antagonists targeting the PDCD1 gene along with immunological antagonists targeting the PD-1/PD-L1 checkpoint is a compelling avenue of research. Such a multifaceted approach may address issues related to an immunological escape and drug resistance that often hinder conventional monotherapies [100].

Platelet-derived growth factor beta (PDGFB) is highly expressed and acts as a molecular marker for drug resistance linked to most mutant ccRCC tumor suppressor genes. Additionally, GITR is renowned for its proinflammatory action, which enhances the proliferation of immune cells. A combination therapy comprising a PDGFB molecular antagonist and a GITR immunological agonist may offer a more efficacious approach for treating ccRCC [96,97,98,99,100,101]. The integration of mTOR antagonists and CTLA4 antagonists with other known ccRCC-specific drug-resistance molecular and immunological biomarker antagonists may yield a more favorable prognostic outcome for patients diagnosed with the high expression of the CTLA4 tumor-promoting biomarker. This is particularly significant due to the recent identification of the coexistence of other tumor-promoting immunological biomarkers with CTLA4 in ccRCC, which facilitates the establishment of a unique immune evasion pathway that counteracts the therapeutic action of anti-CTLA4 monotherapy [96,97,98,99,100,101]. Consequently, the combined use of molecular therapy and specific immunotherapies represents the optimal therapeutic strategy for this specific subgroup of ccRCC patients.

The CD70-CD27 axis plays a critical role in immunological evasion [102]. The suppression of this signaling pathway hampers the growth of malignant kidney tumors. The CD70-CD27 complex, when bound together, primarily targets T-cells for destruction, thus compromising the body’s immune defense and allowing for the proliferation of cancerous cells. It is worth noting that CD27 antagonists are less well understood compared to CD70 antagonists [96,97,98,99,100,101,102,103]. Further investigation is warranted to elucidate this knowledge gap, as the discovery of more effective CD27 antagonists holds great promise for improving immunotherapy for cancerous tumors mediated by the CD70-CD27 axis in ccRCC patients.

PARP inhibitors like olaparib and veliparib weaken the BAP-1 protein’s ability to repair DNA and fight tumors. Using other treatments that do not affect PARP would work better for treating KIRC. BAP-1 is a known tumor suppressor [98,104]. The coexpression of the genetically altered BRAF gene with BAP-1 results in the loss of the genetic integrity of BAP-1. A further empirical study is required to fully comprehend the mechanism underlying this mutation-inducing interaction; nonetheless, theoretically blocking this interaction may enhance the genetic stability of BAP-1 and preserve its tumor-suppressive role.

The phase III clinical trial LITESPARK-005 (NCT04195750) is an important breakthrough in the treatment of advanced Clear Cell Renal Cell Carcinoma (ccRCC), a common type of kidney cancer. This trial studied patients whose cancer had progressed despite being treated with anti-angiogenic therapies (which block the blood supply to tumors) and immune checkpoint inhibitors (which boost the immune system’s ability to fight cancer). It is the first trial to show positive results in this specific group of patients, making it a major step forward in kidney cancer research. One of the key findings of this trial is the effectiveness of a drug called Belzutifan. This drug works by targeting a protein called HIF-2a, which helps cancer cells survive and grow, especially in low-oxygen environments like tumors. By blocking HIF-2a, Belzutifan introduces a new way to treat advanced ccRCC. The results of LITESPARK-005 highlight how important Belzutifan could be as a treatment option for patients in the advanced stages of kidney cancer. For many, current treatments stop working over time, so this drug provides new hope for managing the disease. These findings show that targeting HIF-2a is not just innovative, but could also be life-changing for patients, offering a new approach to improving survival and quality of life in a cancer that is often difficult to treat effectively.

The trial results indicate that both progression-free survival (PFS) and the overall response rate (ORR) experienced statistically significant improvements as a direct consequence of the administration of Belzutifan, showcasing its impressive efficacy [104,105,106]. When compared to the conventional treatment option everolimus, Belzutifan exhibits a remarkable 25% reduction in rates of death and disease progression, highlighting its superiority as a treatment choice. Furthermore, Belzutifan not only proves to be highly effective, but it also exhibits an exceptional level of tolerance in patients, leading to a substantial enhancement in their overall quality of life [104,105,106]. This combination of improved survival outcomes and an increased quality of life positions Belzutifan as an unparalleled and essential treatment option for individuals battling advanced ccRCC. Therefore, it is absolutely crucial for both healthcare professionals and patients to recognize the transformative potential of Belzutifan in the management of advanced ccRCC, as it represents a beacon of hope for those affected by this challenging disease.

Tumorigenesis is potentiated via the oncogenic activities of certain growth factors [107,108], and the development of multifunctional antagonists targeting various ccRCC-implicated growth factors, such as VEGF, PDGFB, and TGFA, could offer a more comprehensive approach to ccRCC therapy. These antagonists may inhibit multiple receptors of key ccRCC-implicated growth factors. Targeting VHL genetic alterations, which upregulate the transcription of growth factors, with a combination of multifunctional antagonists and CD40 agonists could provide a more positive prognostic outcome for ccRCC patients. The idea of blocking the VEGF-induced apoptosis of B and T lymphocytes, while also inhibiting growth factor receptors, represents an innovative approach to ccRCC therapy.

Significant progress has been made in the study of ccRCC transcriptomics, with single-cell RNA sequencing technology emerging as a particularly transformative tool. Single-cell RNA sequencing technology has greatly improved the ability to identify diagnostic, prognostic, and therapeutic biomarkers. This has been particularly helpful in overcoming the challenges posed by the diverse treatment responses seen in patients with metastatic Clear Cell Renal Cell carcinoma (ccRCC), leading to better and more targeted treatments. This technology has also greatly enhanced researchers’ ability to explore and map the intricate interactions that occur between different types of immune and tumor cells in ccRCC. By providing a more detailed and precise view of the cellular microenvironment, single-cell RNA sequencing has uncovered several important mechanisms that underlie the behavior of these cells and their communication networks. However, despite these advances, there remains considerable potential for further exploration and discovery.

Continued improvements in single-cell RNA sequencing technology, along with its wider application, could enable the identification of additional, previously unknown interactions that play a critical role in the progression of ccRCC. These interactions could involve unique pathways or cellular behaviors that drive metastasis, allowing cancer cells to spread to other parts of the body. Furthermore, these advancements may shed light on the mechanisms of drug resistance, helping to explain why some tumors fail to respond to treatments or develop resistance over time. By expanding our understanding of these complex processes, researchers can uncover new therapeutic targets and develop more effective treatment strategies for combating ccRCC.

The use of combination therapy, which pairs tyrosine kinase inhibitors with immunotherapies, has been a major breakthrough in improving how well patients respond to treatment. This approach has brought significant benefits, including better treatment outcomes and higher survival rates for many patients [109,110,111,112,113]. However, one of the biggest challenges limiting the success of this therapy is drug resistance. Studies show that 30–60% of patients experience some level of resistance to these treatments, which reduces their effectiveness and makes it harder to achieve lasting results. This problem remains a serious obstacle despite the promising advancements this therapy has introduced.

To tackle this issue, future research needs to focus on finding new ways to improve treatment. Scientists could look into alternative immune checkpoints, which are different mechanisms in the immune system that could be targeted to enhance the body’s ability to fight disease. Research should also explore new molecular markers, which are specific indicators in the body that can help predict how a patient will respond to treatment. Additionally, there is a need to study metabolic pathways—processes within cells that are not yet fully understood, but could play a role in drug resistance.

By exploring these areas, researchers could develop treatments that are not only more effective, but also less likely to lose their effectiveness over time. This would make combination therapies even more useful and could help a larger number of patients benefit from these advancements. Overcoming drug resistance is critical to improving patient outcomes, ensuring that this innovative approach continues to deliver on its promise.

## 4. Conclusions

HIF1A-HIF2A, mTOR, CD27-CD70, CTLA4, PD-1, PD-L1, and PD-L2 are important biomarkers linked to drug resistance in metastatic Clear Cell Renal Cell Carcinoma (ccRCC). Patients treated with combination therapies such as pembrolizumab with axitinib, avelumab with axitinib, and nivolumab with ipilimumab have shown better results and a better survival outcome compared to those who only received sunitinib. However, it is important to study the long-term safety of these combinations, including potential organ-specific side-effects, in patients with metastatic ccRCC. The future of ccRCC treatment relies on combining molecular and immunological approaches, targeting important biomarkers and pathways, and using technologies like single-cell RNA sequencing to improve precision medicine. Belzutifan and other new combinations also show promising results, but finding ways to overcome the drug resistance associated with them is still a top priority.

## Figures and Tables

**Table 1:** Recently concluded clinical trials of ccRCC combined therapies versus monotherapies using tyrosine kinase inhibitors and immune checkpoint inhibitors.

Trial Number/Follow-Up Period	Therapy	OS (%)	PFS (%)	ORR (%)	CR (%)
Keynote 426 (43 months)	Pembrolizumab and Axitinib (combined therapy)	90	15.1	59.3	5.8
	Sunitinib (monotherapy)	78	11.1	35.7	1.9
Javelin Renal 101 (42 months)	Avelumab and Axitinib (combined)	86	13.8	NA	3.4
	Sunitinib (mono)	83	7.2	NA	1.8
Immotion 151 (36 months)	Atezolizumab and Bevacizumab (combined)	63	11.2	43	9
	Sunitinib (mono)	60	7.7	25	4
Checkmate 214 (80 months)	Nivolumab and Ipilimumab (combined)	60	11.6	42	9
	Sunitinib (mono)	47	8.4	29	1

The Keynote-426 and Javelin Renal 101 trials show improved efficacy and overall survival with pembrolizumab–axitinib and avelumab–axitinib combinations, respectively, compared to sunitinib. Similarly, the Immotion 151 and CheckMate 214 studies demonstrate superior outcomes with atezolizumab–bevacizumab and nivolumab–ipilimumab combinations over sunitinib., OS = overall survival, PFS = progression-free survival, and ORR = objective response rate, NA = not applicable.

**Table 2:** List of ongoing phase III clinical trials of combined ccRCC molecular therapies (tyrosine kinase inhibitors) and immunotherapies (immune checkpoint inhibitors) in a first-line therapy setting.

Trial	Combined Therapy	Reference Therapy	End Result
COSMIC 313 (NCT03937219)	cabozantinib, nivolumab, and ipilimumab	ipilimumab, nivolumab, and placebo	PFS
NCT03793166	cabozantinib, nivolumab, and ipilimumab	nivolumab or nivolumab, and cobazantinib	OS
CheckMate 9ER (NCT03141177)	cabozantinib and nivolumab	sunitinib	PFS
NCT03260894	pembrolizumab and epacadostat	sunitinib or pazopanib	ORR
CLEAR Trial (NCT02811861)	pembrolizumab or everolimus and lenvatinib	sunitinib	PFS
NCT03729245	nivolumab and NKTR-214	sunitinib or cabozantinib	OS ORR
NCT03873402	nivolumab and ipilimumab	nivolumab	PFS ORR

OS = overall survival, PFS = progression-free survival, and ORR = objective response rate.

## Abbreviations

4E-BP1	4E-binding protein 1
APCs	Antigen-Presenting Cells
BAF180	Brahma-related gene 1 Associated Factor 180
BAP-1	BRCA 1 Associated Protein 1
BRAF	B-Raf raf and v-raf murine sarcoma viral oncogene homolog B1
BRCA1	Breast Cancer gene 1
BTLA	B and T Lymphocyte Attenuator
CD134L	Cluster of Differentiation 134 ligand
CD40	Cluster of Differentiation 40
CD70-CD27	Cluster of Differentiation 70-27
CDK1	Cyclin Dependent Kinase 1
chKIRC	Chromophobe Kidney Renal Cell Carcinoma
CR	Complete Response
CTLA4	Cytotoxic T-Lymphocyte Associated 4
CXCL10	C-X-C Motif Chemokine Ligand 10
DCs	Dendritic Cells
DFS	Disease-Free Survival
FH	Fumarate Hydratase
GITR	Glucocorticod-Induced Tumor necrosis factor Receptor
GITRL	Glucocorticod-Induced Tumor necrosis factor Receptor Ligand
HCC-LM3	Hepatocellular Carcinoma Cell Line Metastasis 3
HCF1	Host Cell Factor 1
HIF1A	Hypoxia Inducible Factor 1 Alpha
HIF2A	Hypoxia Inducible Factor 2 Alpha
HIFA	Hypoxia Inducible Factor Alpha
HIFB	Hypoxia Inducible Factor Beta
HVEM	Herpes Virus Entry Mediator
ICAM1	Intracellular Adhesion Molecule
ICI	Immune Check point Inhibitors
KEGG	Kyoto Encyclopedia of Genes and Genomes
ccRCC	Clear Cell Renal Cell Carcinoma (ccRCC)
PRCC	Papillary Renal Cell Carcinoma
mTOR	Mammalian Target of Rapamycin
mTORC1	mTOR-complex1
mTOR-P13K	mTOR Rapamycin Phosphatidylinositol-3-Kinase
NK	Natural Killer cell
